# Intestinal and hepatic effects of iron oxide nanoparticles

**DOI:** 10.1007/s00204-020-02960-7

**Published:** 2021-02-08

**Authors:** Linn Voss, Elisa Hoché, Valerie Stock, Linda Böhmert, Albert Braeuning, Andreas F. Thünemann, Holger Sieg

**Affiliations:** 1 German Federal Institute for Risk Assessment (BfR), Max-Dohrn-Straße 8-10, 10589 Berlin, Germany; 2 German Federal Institute for Material Research and Testing (BAM), Unter den Eichen 87, 12205 Berlin, Germany

**Keywords:** Nanoparticles, Toxicity, Iron oxide nanoparticles, Uptake and transport, Cellular effects

## Abstract

Iron oxide nanoparticles gain increasing attention due to their broad industrial use. However, safety concerns exist since their effects on human cells are still under investigation. The presence of iron oxide nanoparticles in the food pigment E172 has been shown recently. Here, we studied four iron oxide nanoparticles, one food pigment E172 and the ionic control FeSO_4_ regarding dissolution in biological media, uptake and transport, and cellular effects in vitro in human intestinal Caco-2 and HepaRG hepatocarcinoma cells. The iron oxide nanoparticles passed the gastrointestinal passage without dissolution and reached the intestine in the form of particles. Minor uptake was seen into Caco-2 cells but almost no transport to the basolateral site was detected for any of the tested particles. HepaRG cells showed higher particle uptake. Caco-2 cells showed no alterations in reactive oxygen species production, apoptosis, or mitochondrial membrane potential, whereas two particles induced apoptosis in HepaRG cells, and one altered mitochondrial membrane potential at non-cytotoxic concentrations. No correlation between physicochemical particle characteristics and cellular effects was observed, thus emphasizing the need for case-by-case assessment of iron oxide nanoparticles.

**Supplementary Information:**

The online version contains supplementary material available at 10.1007/s00204-020-02960-7.

## Introduction

Iron oxide nanoparticles are widely used chemicals. They can be found in the biomedical sector as well as in the food pigment E172 (Vangijzegem et al. 2019; Voss et al. 2020a). Moreover, they were suggested to be suitable for the fortification or iron supplementation of foods: iron oxide nanoparticles are believed to cause less gastrointestinal side effects than ferrous sulphate or gluconate, fewer changes to food texture and taste, and may have a higher bioavailability than their bulk counterparts (Hilty et al. 2010; Hosny et al. 2015; Tolkien et al. 2015). However, the growing use of iron oxide nanoparticles has raised concerns regarding their safety and data on their toxicological potential are still incomplete. Especially uptake, distribution and toxic effects still require investigation.

Nanoparticles can be highly influenced by their chemical environment. When they are taken up orally, they travel through the gastrointestinal passage, where they are in contact with different fluids, varying in pH values and composition. Other nanoparticles such as zinc oxide, silver, and aluminum are strongly affected by the digestion procedure (Kästner et al. 2017; Sieg et al. 2017; Voss et al. 2020b), whereas data about physico-chemical changes during the digestive process are lacking. Especially the acidic stomach juice may lead to dissolution of the nanoparticles and thereby a release of ionic iron. This ionic iron interacts differently with the cellular machinery; uptake and transport mechanisms can differ tremendously between particles and dissolved ions. While divalent iron ions can be taken up via the Divalent Metal Transporter 1 (DMT1), nanoparticles containing iron are believed to be taken up by endocytosis (Zanella et al. 2017). Moreover, many studies investigating the effect of iron oxide nanoparticles on human cells are conducted in vitro. Therefore, the nanoparticles are dispersed in cell culture medium. To ensure the suitability of the test system, it is decisive to know the ion release of iron oxide nanoparticles in the respective medium compared to the ion release in the digestive tract.

When taken up orally, the intestinal wall plays a crucial role in the systemic uptake of nanoparticles. Even though animal studies have shown that iron levels rise in downstream organs such as the liver after oral treatment with iron oxide nanoparticles, their effect on hepatic and intestinal cells is still not understood (Singh et al. 2013; Chamorro et al. 2015; Garcia-Fernandez et al. 2020). Cellular endpoints such as apoptosis, mitochondrial integrity, and oxidative stress levels can be studied to better understand the impact of nanoparticles on humans, thereby contributing to a reduction of animal sacrifices. Human Caco-2 and HepaRG cells can serve as suitable cell models for the intestinal wall and the liver, respectively (Artursson and Karlsson 1991; Gripon, Rumin et al. 2002). HepaRG cells differentiate into biliary-like and hepatocyte-like cells when treated with dimethyl sulfoxide (DMSO) for 2 weeks (Cerec et al. 2007). Caco-2 cells, originally derived from a colon carcinoma, differentiate during 3 weeks into an intact enterocyte-like cell layer, including tight junctions and microvilli (Artursson and Karlsson 1991; Lichtenstein et al. 2016). Caco-2 cells can be used in transwell systems to imitate the intestinal barrier and investigate uptake and transport mechanisms in the intestine.

When nanoparticles interact with cells of the body, they may alter their homeostasis on various levels. It has been shown that iron oxide nanoparticles can impair cellular homeostasis (Wu et al. 2010; Cromer Berman et al. 2013; Strugari et al. 2019). However, available data are not coherent and there are still knowledge gaps regarding correlation between physicochemical particle characteristics and cellular effects.

In this work, we studied the intestinal and hepatic effects of four iron oxide nanoparticles, one food pigment sold as E172, and soluble FeSO_4_. The kind of nanoparticles used in this study can be found in different E172 pigments, and therefore oral exposure to such particles is likely to occur (Voss et al. 2020a). Ion release was investigated during the digestive cascade as well as in different fluids used in cell culture studies. Uptake and transport at the intestinal barrier were investigated with Caco-2 cells, while hepatic uptake was studied in HepaRG cells. Moreover, various toxicological tests were conducted both in Caco-2 and in HepaRG cells to understand the influence of iron oxide nanoparticles on intestinal and hepatic cells.

## Materials and methods

### Chemicals and nanoparticles

Chemicals were purchased from Sigma-Aldrich (Taufkirchen, Germany), Merck (Darmstadt, Germany), or Carl Roth (Karlsruhe, Germany) if not otherwise indicated. The commercial food dye E172 was purchased from a company in the United Kingdom, the brand of the food pigment is known to the authors and available upon request.

Three nanoparticle dispersions were purchase from US Nano (Huston, TX, USA) containing (1) rod-shaped and spherical particles of γ-Fe_2_O_3_ (15%, orange), (2) rod-shaped particles of α-Fe_2_O_3_ (20%, red) and spherical particles of Fe_3_O_4_ (20%, black). Rod-shaped, yellow γ-Fe_2_O_3_ nanoparticles were obtained from Merck as 20% dispersion. All particles were dispersed freshly at a concentration of 2.56 mg/mL according to the modified NanoGenoTox protocol: Ultrasonication was carried out with a Sonoplus HD 2200 Homogenisator equipped with an apex KE76 (Bandelin, Berlin, Germany) for 5:09 min at 20% amplitude. 15.36 mg E172 was pre-wetted with 60 µL 70% ethanol before addition of water (to a concentration of 2.56 mg/mL) and ultrasonication. For stabilization, BSA was added to all samples to a final concentration of 0.05% immediately after sonication and before use.

### Solubility

To assess solubility in different media, all nanoparticles and FeSO_4_ were diluted in MilliQ H_2_O, and in the cell culture media Dulbecco's Modified Eagle Medium (DMEM; GE Healthcare, Freiburg, Germany) or William’s E Medium. These media resembled the media used in cell culture assays (see below). Incubation was carried out for 24 h at 37 °C. After that, solutions were ultracentrifuged at 100,000×*g* and the supernatant was analyzed for iron content. Atomic Absorption Spectroscopy (AAS) operating in iron mode (PinAAcle 900Z, Perkin Elmer, Singapore, the program can be found in the supplementary material, Table S1).

### In vitro digestion

Artificial in vitro digestion is an established method to assess the influence of digestive juices on different materials (Kästner et al. 2017; Sieg et al. 2017; Stock et al. 2020; Voss et al. 2020b). It consists of three different artificial digestive juices, namely saliva, gastric, and intestinal fluid; their composition can be found in the supplementary material (Table S2).

All nanoparticles, the E172 food pigment and FeSO_4_ were added to the saliva in a concentration of 330 µg Fe/mL. The model resembles the digestion process, adding gastric and intestinal juice subsequently, leading to a dilution of the particles. It started with 16 mL synthetic saliva for 5 min. After that, 10 mL were taken for further analysis. 14 mL of gastric juice were added, and the solution was set to pH 2.0 using hydrochloric acid. After 2 h, another 10 mL was taken, and 10 mL of intestinal fluid was added. The pH was adjusted to 7.5 using sodium bicarbonate and the mixture was stirred for 2 h. In all stages, the fluids were constantly stirred in a water bath at 37 °C. To ensure enzyme activities in the digestive juices, enzyme activity assays were performed and measured photometrically: amylase activity in saliva was verified using amylopectin azure, the activity of pepsin in gastric juice by an albumin/bromophenol blue complex, lipase activity using 4-methylumbelliferyl oleate, and tryptic activity was confirmed using azocasein as substrates, respectively.

### Cell cultivation

HepaRG cells (Biopredic HPR101, St. Gregoire, France) were cultivated according to the recommended protocol using William’s E Medium supplemented with 10% (v/v) FCS, 100 U/mL penicillin and 100 μg/mL streptomycin (P/S), 5 μg/mL insulin and 5 × 10^−5^ M hydrocortisone hemisuccinate as published before (Luckert et al. 2017; Sieg et al. 2019) HepaRG cells were cultivated for 2 weeks without supplementing DMSO and for another 2 weeks with supplementing 1.7% DMSO. For incubation, the cells were adjusted to the assay medium with 0.5% DMSO and 2% FCS 2 days prior to incubation. On the day of incubation with nanoparticles, the test substances were diluted in assay medium.

Caco-2 cells (ECACC: 86010202) were obtained from the European Collection of Authenticated Cell Cultures (Salisbury, UK) and cultivated in DMEM with 10% FCS and 105 Units/L penicillin and 100 μg/mL streptomycin. For all assays, Caco-2 cells were differentiated for 21 days.

Both cell lines were kept at 37 °C and 5% CO_2_ in a humidified atmosphere. After seeding in the appropriate plate format, cells were fed every 2–3 days. For incubation, cell culture medium was replaced by 100 µL of the different particles suspended in cell-specific phenol red-free medium.

### Uptake and transport

50,000 Caco-2 cells were seeded in 12-well transwell plates with inserts of 1.12 cm^2^ growth area and a 3 µm pore size polycarbonate membrane (Corning Incorporated, New York City, NY, USA) and differentiated for 3 weeks. Membrane integrity was checked by transepithelial electrical resistance (TEER) with an EVOM2 Electrode (World Precision Instruments, Sarasota, FL, USA). After incubation, the apical and basolateral media were collected, the inserts were washed, and the membrane was cut out for further analysis. All compartments were analyzed by AAS as described above.

HepaRG cells were seeded in 12-well plates and differentiated for 4 weeks as described above. After incubation, the supernatant was collected, and cells were washed with PBS. Cells were trypsinized and collected. Supernatant, wash fractions and cells were analyzed for iron content by AAS as described above.

### Cellular effects

HepaRG and Caco-2 cells were seeded in 96-well plates at a density of 9000 (Hepa-RG) or 5000 (Caco-2) cells per well. They were cultivated for 4 (HepaRG) or 3 (Caco-2) weeks as described above. Test substances were diluted in the depicted concentrations in appropriate assay medium and cells were incubated for 24 h in a volume of 100 µL. For all assays, color interference was taken into account by measuring wells with particles but without cells and calculating this into the background signal.

Cell viability was assessed using the 3-(4,5-dimethylthiazole-2-yl)-2,5-diphenyltetrazolium bromide (MTT) assay for mitochondrial function according to the manufacturer’s instructions (Merck, Darmstadt, Germany). 0.01% Triton X-100 served as a positive control. Mitochondrial membrane potential was investigated using the JC-10 assay, which is based on a fluorescent dye that is red in mitochondria but turns green after release into the cytosol due to depolarization of the mitochondrial membrane. 2 h before end of incubation, the positive control 1 mg/mL verapamil was added to the designated cells. After incubation, all media with test substances was replaced with 50 µL of 50 µM verapamil for 15 min. Then, verapamil was removed and 100 µL of 10 µM JC-10 (Santa-Cruz Biotechnologies, Dallas, TX, USA) in phosphate buffered saline (PBS) was added for 30 min (37 °C, 5% CO_2_). Cells were washed with PBS twice and measured in 100 µL PBS at ex./em. 490/525 nm and ex./em. 540/590 nm. The 525 nm/590 nm fluorescence ratio was used to determine the membrane potential. The MCB assay was performed to investigate reduced glutathione levels. After 24 h of treatment, the test substances were removed and 250 μL MCB solution (40 μM) was added for 30 min at 37 °C and 5% CO_2_. After washing with PBS, cells were lysed with the desorption solution described for MTT and fluorescence was measured (ex./em.380/ 460 nm). Buthionine sulfoximine (BSO) (100 μM) served as a positive control.

All assays were measured on a multi-well plate reader (Tecan, Männedorf, Switzerland).

Activities of caspase 3, caspase 8 and caspase 9 were measured using specific substrates, which are cleaved by the enzymes into fluorescent products. After 24 h of incubation, particles were removed and 50 µL lysis buffer (50 mM HEPES, 2% v/v Triton X-100, pH 7.4) was added. Plates were shaken for 15 min until cell debris was visible. Then, 100 µl of reaction buffer (50 mM HEPES, 5 mM EDTA, 0.1% (v/v) CHAPS, 5% (v/v) glycerin (pH 7.4)) containing 50 µM of the respective substrates and 5 µM dithiothreitol (DTT) was added per well. The substrates were: Ac-DEVD-AFC (caspase 3), Ac-IETD-AFC (caspase 8) and Ac-LEHD-AFC (caspase 9) (all from Cayman Chemical, Ann Arbor, MI, USA). After 5 h, plates were measured at ex./em. 400/505 nm (caspase 9) or at ex./em. 380/500 nm (caspase 3, caspase 8). For Western blotting analysis, cells were differentiated in six-well plates and incubated with iron oxide nanoparticles for 24 h. After that, cells were washed three times with ice-cold PBS containing protease inhibitor cocktails and lysed using RIPA buffer (50 mM Tris–HCl, 150 mM NaCl, 2 µM EGTA, 0.1% (w/v) SDS, 0.5% (w/v) deoxycholic acid) supplemented with protease inhibitor (Roche, Basel, Switzerland). After cell lysis, protein content was assessed with the BSA assay and 20 µg protein was used for Western-Blot analysis. Therefore, a 12% SDS-PA-gel was used. Protein transfer was achieved by semi-dry blotting technique. After blotting, the membrane was stained with ponceau red to ensure effective protein transfer and blocked with 5% milk powder in PBS. Cleaved caspases were detected using primary rabbit antibodies (#9661S, #7237S, #52873, in 1:1000 dilution, Cell signaling Technology, Frankfurt am Main, Germany) and secondary Goat-anti-rabbit HRP (#HAF008, 1:5000 dilution, R&D Systems, Minneapolis, USA). Pan-actin served as loading control and was detected with pan-actin mouse mAb and sheep anti-mouse HRP (#MS-1295-P, 1:1000 dilution, Thermo Fisher Scientific, CA, USA, and #A-014HRP, 1:5000 dilution, Serum Diagnostica, Heidesee, Germany).

To assess the ATP-content of cells, the commercial CellTiter-Glo Luminescent Cell Viability Assay kit from promega (Walldorf, Germany) was used according to the manufacturer’s instructions with 1 µM oligomycin as positive control. To control for interference with the particles, particle-containing assay medium was given in 96-well plates without cells. 100 µl ATP-assay solution was added as well as 1 µM ATP. The luminescence signal was read in 30 min intervals for 5 h. This assay was only done with HepaRG cells, because of interference when Caco-2 cells were used.

## Results

### Characteristics of nanoparticles

All nanoparticles were analyzed in detail in our recent publication (Voss et al. 2020c). The results are summarized in Table 1. As in our previous publication, we assigned symbols to each particle: rod-shaped α-Fe_2_O_3_ is symbolized as a red rod, rod and spherical γ-Fe_2_O_3_ as an orange rod and sphere, spherical γ-Fe_2_O_3_ as a yellow rod, and spherical Fe_3_O_4_ as a black sphere. The E172 pigment in this study has been characterized thoroughly in our previous study (Voss et al. 2020a).

**Table 1:** Summary of iron oxide nanoparticle characterization

Nanomaterial	Fe_2_O_3_ alpha	Fe_2_O_3_ gamma	Fe_2_O_3_ gamma	Fe_3_O_4_
Shape	Rods	Spheres + rods	Rods	Spheres
Symbol				
TEM-picture				
Mean by TEM [nm]	L:41, W:7	S:124, L:99, W:13	L:44, W:10	S:11
Z-average by DLS (PDI)	89 (0.3)	294 (0.4)	112 (0.4)	163 (0.3)
Mean by spICP-MS [nm]	L:111, W:19	S: 48, L:163, W:21	L:88, W:20	S: 39

Iron oxide nanoparticles were characterized using TEM, DLS, and spICP-MS. L = length, W = width, S = square diameter, TEM and spICP-MS data in nm. Detailed information can be found elsewhere (Voss et al. 2020a, b, c)

### Solubility of iron oxide nanoparticles

**Fig. 1:**
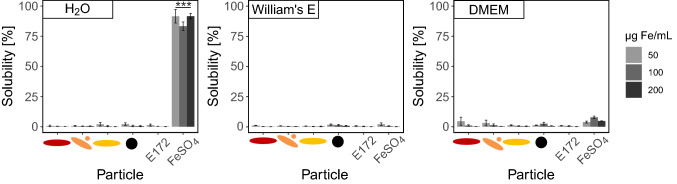
Solubility of iron oxide nanoparticles in different media. Iron oxide nanoparticles, E172 and FeSO_4_ were dispersed in MiliQ H_2_O, William’s E, and DMEM cell culture medium in concentrations from 50 to 200 µg Fe/mL and incubated for 24 h. Ion release was assessed using ultracentrifugation and iron measurements in the supernatant with AAS. Mean ± SD, *n* = 3, statistical analysis was done with one-way ANOVA followed by Dunnett’s test (*p < 0.05, **p < 0.01, ***p < 0.001)

Ion release was assessed in different media as well as during the individual steps of the in vitro digestion procedure to ensure the suitability of in vitro cell culture experiments with respect to the dissolution behavior of iron oxide nanoparticles. The particles display a low solubility (below 5%) in H_2_O, Willliams’s E, and DMEM medium in concentrations between 50 and 200 µg Fe/mL (see Fig. 1). Only FeSO_4_ dissolved in H_2_O to about 100% but was present nearly insoluble in William’s E and DMEM. In DMEM, a slightly higher dissolution was seen for FeSO_4_ (7.8% at 100 µg Fe/mL) but this was not significantly different from the other samples or the background (cell culture medium only). No significant differences in solubility were found for the range of iron concentration used in this study.

### In vitro digestion

**Fig. 2:**
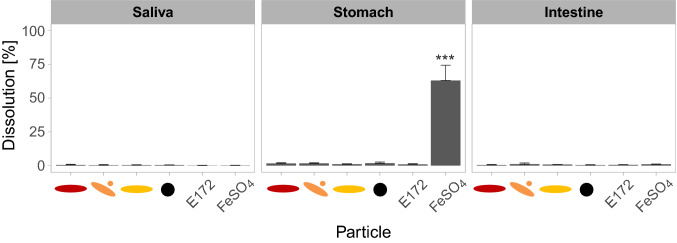
Ion release of iron oxide nanoparticles during in vitro digestion. Supernatants of centrifuged samples were analyzed regarding their ionic iron content. Depicted are results for artificial saliva, gastric juice and intestinal juice with 50 µg Fe/mL in the intestinal juice. Mean ± SD, *n* = 3, statistical analysis was done with one-way ANOVA followed by Dunnett’s test (*p < 0.05, **p < 0.01, ***p < 0.001)

All particles including the food pigment behaved very similar in the in vitro digestion (Fig. 2). Almost no ion release was observed in the saliva, the stomach, and the intestine (5% or lower). Ion release was calculated with respect to the total number of iron atoms available from the iron oxide nanoparticles. FeSO_4_ showed dissolution in the acidic stomach to about 60%, but formed particles in the saliva and reformed particles in the intestinal juice. The dissolution behavior of all iron species in the intestinal juice was similar to the dissolution in cell culture medium.

### Uptake and transport

**Fig. 3:**
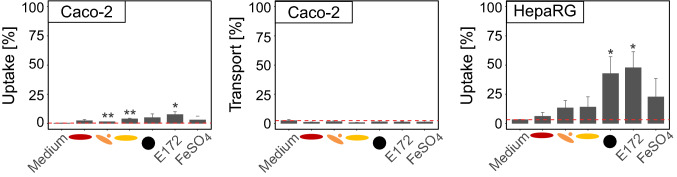
Uptake and transport of iron oxide nanoparticles in Caco-2 and HepaRG cells. For Caco-2 cells, uptake and transport could be assessed using a transwell system. Therefore, differentiated Caco-2 cells were treated with 50 µg Fe/mL for 24 h. Cells were washed and the membrane was collected for uptake analysis while the iron content in the basolateral compartment indicated transport. Cells were differentiated in a 12-well system and treated with 50 µg Fe/ml for 24 h. After that, the cell layer and the supernatant were analyzed for iron content separately. For HepaRG cells, only iron uptake was investigated. The red dashed line indicates the background resulting from the iron content in the medium control plus the limit of quantification. Mean ± SD, *n* = 3, statistical analysis was done with one-way ANOVA followed by Dunnett’s test (*p < 0.05, **p < 0.01, ***p < 0.001)

To better understand the interaction of iron oxide nanoparticles with Caco-2 and HepaRG cells, uptake and transport (in case of Caco-2 cells) were investigated. Results are shown in Fig. 3. To assess background iron levels, a medium control was analyzed. Both γ-Fe_2_O_3_ and the E172 sample showed significantly higher uptake in Caco-2 cells than the medium control. However, the overall uptake was rather low, with 1.41% (rod and spherical γ-Fe_2_O_3_), 3.83% (rod γ-Fe_2_O_3_), and 7.53% (E172). Fe_3_O_4_ showed ca. 4% uptake, but the obtained value was not significantly different from the background. Transport to the basolateral side of the transwell was not detectable for all iron species investigated. On the other hand, HepaRG cells showed a more pronounced uptake of all but the α-Fe_2_O_3_ particle. However, this was only significant for Fe_3_O_4_ and E172 with 42.7 and 47.7% uptake, respectively. Of the γ-Fe_2_O_3_-particles, between 13 and 14% were found in the cellular layer, and 22.7% of iron from FeSO_4_ was detected with HepaRG cells.

### Cellular effects

**Fig. 4:**
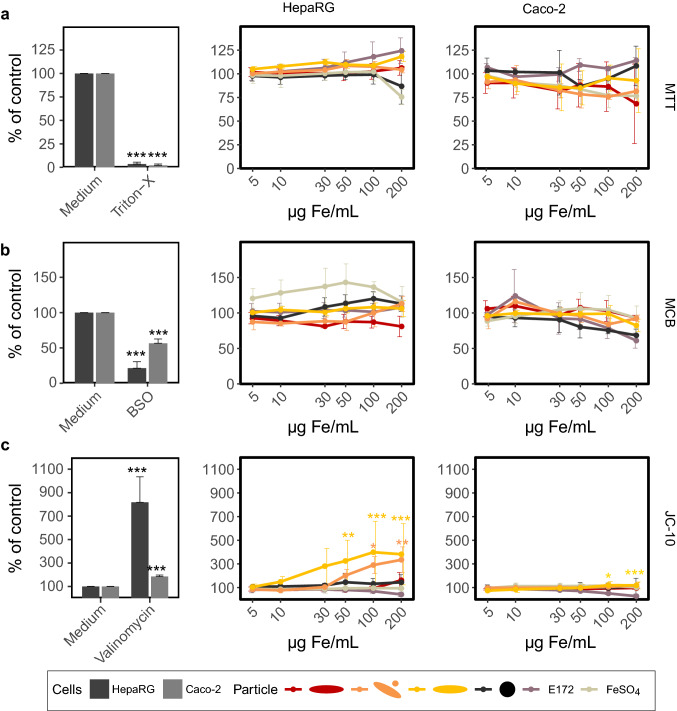
Cellular effects of iron oxide nanoparticles on HepaRG and Caco-2 cells. Both cell lines were treated with increasing concentrations of nanoparticles, E172, and FeSO_4_ for 24 h. After that, different tests were conducted to investigate **a** mitochondrial activity using the MTT assay, **b** reduced glutathione-status with the MCB assay, and **c** mitochondrial membrane potential with the JC-10 assay. Triton X-100, BSO, and valinomycin served as a positive control for the MTT, MCB, and JC-10 assay, respectively. Mean ± SD, *n* = 3, statistical analysis was done with one-way ANOVA followed by Dunnett’s test (*p < 0.05, **p < 0.01, ***p < 0.001)

**Fig. 5:**
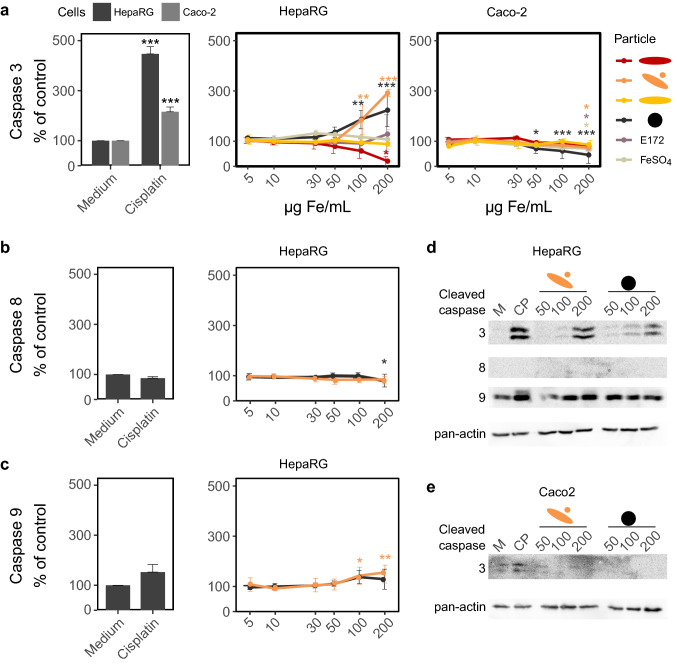
Influence of iron oxide nanoparticles on caspase activities in HepaRG and Caco-2 cells. **a** Caspase 3 activity was assessed in HepaRG and Caco-2 cells. Since caspase 3 was only activated by two iron oxide nanoparticles and only in HepaRG cells, activities of caspases 8 (**b**) and 9 (**c**) were only investigated under these conditions. Mean ± SD, *n* = 3, statistical analysis was done with one-way ANOVA followed by Dunnett’s test (*p < 0.05, **p < 0.01, ***p < 0.001). **d**, **e** Show Western blot analysis of all investigated caspases for HepaRG and Caco-2 cells, respectively

Most of the iron oxide nanoparticles, as well as E172 and FeSO_4_ showed only minor effects on Caco-2 and HepaRG cells (Figs. 4 and 5). In the MTT assay, cell viability was stable for all test substances up to 200 µg Fe/mL (Fig. 4a). Similarly, the status of reduced glutathione assessed by the MCB assay did not change remarkably (Fig. 4b). When treated with the two types of γ-Fe_2_O_3_ particles, the mitochondrial membrane potential of HepaRG cells was impaired at high iron concentrations (Fig. 4c). This effect was not seen in Caco-2 cells. E172 led to a decreased signal ratio in the JC-10 assay in Caco-2 cells, which could indicate a hyperpolarization of the cells. However, since this signal only occurred at 200 µg Fe/mL, where cells are covered with colorful particles, it is not supposed to be a sign of cellular impairment but rather an interference of the particles with the assay. Such an interference has already been seen with other assays and nanoparticles, especially the also used ATP assay here (Zitat Lehmann, 10.1016/j.ab.2016.03.019). Additionally, it is possible that the reagent does not reach the cells sufficiently through the physical barrier. To assess whether the impairment of mitochondrial membrane potential of HepaRG cells after treatment with the γ-Fe_2_O_3_ particles resulted in decreased ATP-levels, ATP contents in HepaRG cells were assessed. The ATP content of HepaRG cells was stable up to 200 µg Fe/mL with all tested substances. Only FeSO_4_ led to a small but insignificant decrease at 200 µg Fe/mL (supplementary material, Figure S1).

Increased activity of the executioner caspase 3 was found in HepaRG cells only, and only after treatment with the spherical and rod-shaped γ-Fe_2_O_3_ and the Fe_3_O_4_ particles (Fig. 5 a). The decreased activity of caspase-3 in Caco-2 cells (around 80%) after treatment with FeSO_4_, the rod and spherically shaped γ-Fe_2_O_3_ and E172 at 200 µg Fe/mL was statistically significant. However, this decrease is probably due to interference of the high amounts of particles with the assay. In this concentration, the cells are covered in particles and it is very difficult to transfer aliquots without particles to the measurement plate. The same is true for Fe_3_O_4_ in Caco-2 cells and α-Fe_2_O_3_ particles in HepaRG cells. Therefore, the activity of the upstream caspases 8 and 9 was only assessed in HepaRG cells with spherical and rod-shaped γ-Fe_2_O_3_ and the Fe_3_O_4_ particles (Fig. 5b, c). There, an increase was shown for caspase 9 activity at high concentrations (100 and 200 µg Fe/mL) after treatment with spherical and rod-shaped γ-Fe_2_O_3_, Fe_3_O_4_ led also to a slight increase, but this was not significant. Western blot analysis of cleaved caspases confirmed these results (Fig. 5d, e).

In the same manner, the ratio of apoptotic and necrotic cells did not change significantly for HepaRG and Caco-2 cells when treated with 50, 100, and 200 µg Fe/mL (see supplementary material, Figure S2). Also, anti-apoptotic genes rather than pro-apoptotic genes were upregulated, especially *BCL-2* in HepaRG cells (see supplementary material, Figure S3).

## Discussion

Even though nanoparticle use has increased in the twenty-first century, risk assessment of these materials is still difficult. As for most nanoparticles, data for risk evaluation of iron oxide nanoparticles are incomplete. Knowledge gaps exist especially regarding the interaction with the gastrointestinal and hepatic system.

Iron oxides are almost insoluble in water and at neutral pH (Cornell 2003). However, it was not known how they interact with the solutions of the gastrointestinal tract, where especially the acidic stomach juice could lead to higher ion release. Here, we showed that iron oxide nanoparticles remain mostly unaltered by the digestive fluids. This is different to other nanoparticles investigated with the in vitro digestion system (Böhmert et al. 2014; Stock et al. 2020; Voss et al. 2020b). Since iron oxide nanoparticles show similar physicochemical properties in the simulated digestive fluid and in tested cell culture media, in vitro cell culture studies are suitable to study the cellular effects of the particles. This is also true for soluble FeSO_4_, even though it dissolved completely in the acidic stomach juice. In the final stage of the in vitro digestive cascade, the intestinal fluid, FeSO_4_ showed a de novo formation of particles, similar to the behavior in DMEM and William’s E medium.

The Caco-2 transwell system provides an in vitro model to determine uptake and transport of xenobiotics at the intestinal barrier. Here, it was shown that iron oxide nanoparticles are taken up to small extent by Caco-2 cells, whereas no transport to the basolateral department was detected. This has also been shown by others using the Caco-2 transwell system either in monoculture or in combination with HT-29-MTX or goblet cells (Kenzaoui et al. 2012; Strugari et al. 2019). However, increased iron levels in spleen and liver were found after oral exposure to iron oxide nanoparticles in in vivo studies, indicating that some particles can cross the intestinal barrier (Singh et al. 2013; Chamorro et al. 2015; Garcia-Fernandez et al. 2020). This discrepancy may be due to the morphology of the Caco-2 cell model, which shows stronger tight junctions than the intestinal barrier in vivo (Hilgendorf et al. 2000). Moreover, the intestinal barrier is composed of different cell types. Co-culture models aim to better represent the in vivo situation, but to date it has not been possible to reproduce the complex interactions of epithelial cells, immune cells and the microbiome in vitro.

HepaRG cells showed stronger interactions with the iron oxide nanoparticles tested here. Especially for the Fe_3_O_4_ particle and E172, uptake was with almost 50% very high. Even though the uptake of the γ-Fe_2_O_3_ particles and FeSO_4_ was not significant in HepaRG cells, the interaction between the test substances and HepaRG cells were higher than in Caco-2 cells. The only particle that showed almost no uptake was the α-Fe_2_O_3_ particle. This is in line with our previous publication, where we studied the influence of iron oxide nanoparticles on the xenobiotic metabolism of HepaRG cells, in which the α-Fe_2_O_3_ particle exhibited the smallest effect of all tested particles (Voss et al. 2020c). However, due to limitations of the AAS method, it is difficult both for HepaRG and Caco-2 cells to distinguish between an interaction of the particles with the cell surface and an internalization of the particles. Strugari et al. stated that the measured uptake of iron oxide nanoparticles in a Caco-2/HT29-MTX model may rather be aggregates of iron oxide nanoparticles on the cell surface (Strugari et al. 2019). Nevertheless, the aggregates could also lead to cytotoxic effects as suggested by Soto et al. (Soto et al. 2007). HepaRG cells develop a more porous cell layer than Caco-2 cells in a transwell system, in which nanoparticles could be embedded better, thus impeding the removal of loosely attached particles by washing steps. Despite extensive washing before collecting the cell layer, it cannot be excluded that the iron in Fig. 3 is not situated inside HepaRG cells but on their surface. On the other hand, in vivo studies showed a strong persistence of iron oxide nanoparticles or their breakdown products in the liver, suggesting a strong uptake and interaction of iron oxide nanoparticles by hepatic cells (Briley-Saebo et al. 2004; Levy et al. 2011). Several studies suggest different uptake mechanisms of iron oxide nanomaterials, with endosomal-lysosomal uptake being one of the most prominent ones (Kenzaoui et al. 2012; Khalid et al. 2018). However, exact localization of the particle on or within the cell is very difficult when no fluorescent labeling is used, since single particles can hardly be detected. Moreover, detection of iron alone is not sufficient to determine if the iron oxide nanoparticle is located within a specific organelle. The often-applied Prussian blue staining for example does not distinguish between different iron forms, e.g., particle, protein bound or ionic, so that it cannot be determined if the iron enters the cell in particle form or if only solubilized iron is taken up. Therefore, better methods are needed to pinpoint exact localizations of nanoparticles on and within cells to better understand their interaction.

The stronger interaction of the particles with liver cells is also reflected in the cellular assays. While iron oxide nanoparticles did not show any adverse effect in any assay, some iron oxide nanoparticles influenced mitochondrial membrane potential as well as apoptosis in HepaRG cells. Thereby, the α-Fe_2_O_3_ particle exhibited the smallest effect in all assays. However, even though the rod-shaped γ-Fe_2_O_3_ particle impaired mitochondrial membrane potential, no effect was seen on the ATP level. This is in line with results from Baratli et al. who treated male Wistar rats with increasing concentrations of iron oxide nanoparticles and did not detect any changes in mitochondrial respiratory chain complex activities and coupling (Baratli et al. 2013). Moreover, also Hussain et al. did not see any changes in mitochondrial membrane potential after treatment of BRL 3A rat liver cells with up to 50 µg Fe/mL (Hussain et al. 2005). In the same manner, Fe_3_O_4_ and the rod- and spherically shaped Fe_2_O_3_ particle induced caspase 3 and 9 activities, increasing intrinsic apoptosis, but neither cell viability nor the ratio of apoptotic, necrotic, and viable cells, as determined by flow cytometry analysis, were altered (supplementary material, Figure S2). It has to be noted, that here, only one time point was investigated (24 h) and that apoptotic events could be initialized after that. However, in our previous study we did not see any changes in cell viability even after 48 h of exposure (Voss et al. 2020c). Moreover, the FACS-results and the gene expression analysis do not indicate signs of early apoptosis (supplementary material, Figure S2 and S3). Similar results have been reported by Zhu et al. (2010). Even though these authors reported different cellular effects like impaired membrane potential and increased ROS levels, the cytotoxic effects were still minor. When we increased the incubation time to 48 h, we still did not observe any cytotoxic effects (Voss et al. 2020c). The results of the qRT-PCR suggest that treatment with nanoparticles leads to an upregulation of anti-apoptotic genes such as *BCl-2 *and a downregulation of pro-apoptotic genes such as *BAD* and *DIABOLO* (see supplementary material, Figure S3). This could prevent increased cell death despite slightly increased caspase activity. However, the exact mechanisms, how some iron oxide nanoparticles lead to cellular impairment is still under investigation and more research is needed to understand how they interfere with the cellular machinery.

## Conclusion

In summary, we showed that iron oxide nanoparticles interact differently with HepaRG and Caco-2 cells. While there was almost no uptake or transport in Caco-2 cells, the interactions with HepaRG cells were very strong for some particles. Overall, the intestinal Caco-2 cells were impaired only slightly by all investigated iron oxide nanoparticles. Even though HepaRG cells showed higher caspase activities after treatment with two tested particles, no significant increase of cytotoxicity could be detected, suggesting anti-apoptotic cross signaling. For the minor effects observed, no clear dependency on size, shape or chemical structure could be drawn, in line with our previous findings. These results emphasize the difficulty of grouping approaches in nanotoxicology and suggest case-by-case approaches in risk assessment of iron oxide nanoparticles.

## Supplementary Information

Below is the link to the electronic supplementary material.

Supplementary file1 (DOCX 309 KB)

## References

[CR1] Artursson P, Karlsson J (1991). Correlation between oral-drug absorption in humans and apparent drug permeability coefficients in human intestinal epithelial (Caco-2) cells. Biochem Biophys Res Commun.

[CR2] Baratli Y, Charles AL, Wolff V, Ben Tahar L, Smiri L, Bouitbir J, Zoll J, Piquard F, Tebourbi O, Sakly M, Abdelmelek H, Geny B (2013). Impact of iron oxide nanoparticles on brain, heart, lung, liver and kidneys mitochondrial respiratory chain complexes activities and coupling. Toxicol In Vitro.

[CR3] Böhmert L, Girod M, Hansen U, Maul R, Knappe P, Niemann B, Weidner SM, Thünemann AF, Lampen A (2014). Analytically monitored digestion of silver nanoparticles and their toxicity on human intestinal cells. Nanotoxicology.

[CR4] Briley-Saebo K, Bjornerud A, Grant D, Ahlstrom H, Berg T, Kindberg GM (2004). Hepatic cellular distribution and degradation of iron oxide nanoparticles following single intravenous injection in rats: implications for magnetic resonance imaging. Cell Tissue Res.

[CR5] Cerec V, Glaise D, Garnier D, Morosan S, Turlin B, Drenou B, Gripon P, Kremsdorf D, Guguen-Guillouzo C, Corlu A (2007). Transdifferentiation of hepatocyte-like cells from the human hepatoma HepaRG cell line through bipotent progenitor. Hepatology.

[CR6] Chamorro S, Gutierrez L, Vaquero MP, Verdoy D, Salas G, Luengo Y, Brenes A, Jose Teran F (2015). Safety assessment of chronic oral exposure to iron oxide nanoparticles. Nanotechnology.

[CR7] Cornell RMSU (2003). The iron oxides: structure, properties, reactions, occurrences and uses.

[CR8] Cromer Berman SM, Kshitiz, Wang CJ, Orukari I, Levchenko A, Bulte JWM, Walczak P (2013). Cell motility of neural stem cells is reduced after SPIO-labeling, which is mitigated after exocytosis. Magn Reson Med.

[CR9] Garcia-Fernandez J, Turiel D, Bettmer J, Jakubowski N, Panne U, RivaGarcia L, Llopis J, SanchezGonzalez C, Montes-Bayon M (2020). In vitro and in situ experiments to evaluate the biodistribution and cellular toxicity of ultrasmall iron oxide nanoparticles potentially used as oral iron supplements. Nanotoxicology..

[CR10] Gripon P, Rumin S, Urban S, Le Seyec J, Glaise D, Cannie I, Guyomard C, Lucas J, Trepo C, Guguen-Guillouzo C (2002). Infection of a human hepatoma cell line by hepatitis B virus. Proc Natl Acad Sci USA.

[CR11] Hilgendorf C, Spahn-Langguth H, Regardh CG, Lipka E, Amidon GL, Langguth P (2000). Caco-2 versus Caco-2/HT29-MTX co-cultured cell lines: permeabilities via diffusion, inside- and outside-directed carrier-mediated transport. J Pharm Sci.

[CR12] Hilty FM, Arnold M, Hilbe M, Teleki A, Knijnenburg JTN, Ehrensperger F, Hurrell RF, Pratsinis SE, Langhans W, Zimmermann MB (2010). Iron from nanocompounds containing iron and zinc is highly bioavailable in rats without tissue accumulation. Nat Nanotechnol.

[CR13] Hosny KM, Banjar ZM, Hariri AH, Hassan AH (2015). Solid lipid nanoparticles loaded with iron to overcome barriers for treatment of iron deficiency anemia. Drug Design Dev Therapy.

[CR14] Hussain SM, Hess KL, Gearhart JM, Geiss KT, Schlager JJ (2005). In vitro toxicity of nanoparticles in BRL 3A rat liver cells. Toxicol In Vitro.

[CR15] Kästner C, Lichtenstein D, Lampen A, Thünemann AF (2017). Monitoring the fate of small silver nanoparticles during artificial digestion. Colloids Surf A Physicochem Eng Asp.

[CR16] Kenzaoui BH, Vila MR, Miquel JM, Cengelli F, Juillerat-Jeanneret L (2012). Evaluation of uptake and transport of cationic and anionic ultrasmall iron oxide nanoparticles by human colon cells. Int J Nanomed.

[CR17] Khalid MK, Asad M, Henrich-Noack P, Sokolov M, Hintz W, Grigartzik L, Zhang E, Dityatev A, van Wachem B, Sabel BA (2018). Evaluation of toxicity and neural uptake in vitro and in vivo of superparamagnetic iron oxide nanoparticles. Int J Mol Sci.

[CR18] Levy M, Luciani N, Alloyeau D, Elgrabli D, Deveaux V, Pechoux C, Chat S, Wang G, Vats N, Gendron F, Factor C, Lotersztajn S, Luciani A, Wilhelm C, Gazeau F (2011). Long term in vivo biotransformation of iron oxide nanoparticles. Biomaterials.

[CR19] Lichtenstein D, Meyer T, Böhmert L, Thünemann AF, Estrela-Lopis I, Braeuning A, Lampen A (2016). Quantification of cellular uptake of silver nanoparticles in intestinal cell models of various complexities. Naunyn-Schmiedebergs Arch Pharmacol.

[CR20] Sieg H, Kästner C, Krause B, Meyer T, Burel A, Böhmert L, Lichtenstein D, Jungnickel H, Tentschert J, Laux P, Braeuning A, Estrela-Lopis I, Gauffre F, Fessard V, Meijer J, Luch A, Thünemann AF, Lampen A (2017). Impact of an artificial digestion procedure on aluminum-containing nanomaterials. Langmuir.

[CR21] Singh SP, Rahman MF, Murty US, Mahboob M, Grover P (2013). Comparative study of genotoxicity and tissue distribution of nano and micron sized iron oxide in rats after acute oral treatment. Toxicol Appl Pharmacol.

[CR22] Soto K, Garza KM, Murr LE (2007). Cytotoxic effects of aggregated nanomaterials. Acta Biomater.

[CR23] Stock V, Fahrenson C, Thuenemann A, Donmez MH, Voss L, Böhmert L, Braeuning A, Lampen A, Sieg H (2020). Impact of artificial digestion on the sizes and shapes of microplastic particles. Food Chem Toxicol.

[CR24] Strugari AFG, Stan MS, Gharbia S, Hermenean A, Dinischiotu A (2019). Characterization of nanoparticle intestinal transport using an in vitro co-culture model. Nanomaterials.

[CR25] Tolkien Z, Stecher L, Mander AP, Pereira DI, Powell JJ (2015). Ferrous sulfate supplementation causes significant gastrointestinal side-effects in adults: a systematic review and meta-analysis. PLoS ONE.

[CR26] Vangijzegem T, Stanicki D, Laurent S (2019). Magnetic iron oxide nanoparticles for drug delivery: applications and characteristics. Expert Opin Drug Deliv.

[CR27] Voss L, Hsiao IL, Ebisch M, Vidmar J, Dreiack N, Böhmert L, Stock V, Braeuning A, Loeschner K, Laux P, Thünemann AF, Lampen A, Sieg H (2020). The presence of iron oxide nanoparticles in the food pigment E172. Food Chem.

[CR28] Voss L, Saloga PEJ, Stock V, Böhmert L, Braeuning A, Thünemann AF, Lampen A, Sieg H (2020). Environmental impact of ZnO nanoparticles evaluated by in vitro simulated digestion (vol 3, pg 724, 2020). ACS Appl Nano Mater.

[CR29] Voss L, Yilmaz K, Burkard L, Vidmar J, Stock V, Hoffmann U, Potz O, Hammer HS, Peiser M, Braeuning A, Loschner K, Böhmert L, Sieg H (2020). Impact of iron oxide nanoparticles on xenobiotic metabolism in HepaRG cells. Arch Toxicol.

[CR30] Wu XY, Tan YB, Mao H, Zhang MM (2010). Toxic effects of iron oxide nanoparticles on human umbilical vein endothelial cells. Int J Nanomed.

[CR31] Zanella D, Bossi E, Gornati R, Bastos C, Faria N, Bernardini G (2017). Iron oxide nanoparticles can cross plasma membranes. Sci Rep.

[CR32] Zhu MT, Wang Y, Feng WY, Wang B, Wang M, Ouyang H, Chai ZF (2010). Oxidative stress and apoptosis induced by iron oxide nanoparticles in cultured human umbilical endothelial cells. J Nanosci Nanotechnol.

