# Supplementary material for: Intestinal and hepatic effects of iron oxide nanoparticles
Source: Arch Toxicol. 2021 Feb 8;95(3):895–905. doi: 10.1007/s00204-020-02960-7 (PMC7904561; doi:10.1007/s00204-020-02960-7)
Supplement: Supplementary file 1 — Supplementary file1 (DOCX 309 KB) [file 204_2020_2960_MOESM1_ESM.docx]

Supplementary material: Intestinal and hepatic effects of iron oxide nanoparticles

Linn Voss^1^, Elisa Hoché^1^, Valerie Stock^1^, Linda Böhmert^1^, Albert Braeuning^1^, Andreas F. Thünemann^2^, Holger Sieg^1^

1 German Federal Institute for Risk Assessment (BfR), Max-Dohrn-Straße 8-10, 10589 Berlin, Germany

2 German Federal Institute for Material Research and Testing (BAM), Unter den Eichen 87, 12205 Berlin

Supplementary Table 1 Atomic absorption spectrometry program for iron detection. Samples were measured with a zinc lamp after acidic hydrolysis using a microwave system.

| **Step #** | **Temperatur [°C]** | **Ramp time [sec]** | **Hold time [sec]** | **Internal flow [mL/min]** | **Gas type** |
| --- | --- | --- | --- | --- | --- |
| 1 | 110 | 1 | 30 | 250 | Normal |
| 2 | 130 | 15 | 30 | 250 | Normal |
| 3 | 1400 | 10 | 20 | 250 | Normal |
| 4 | 2100 | 0 | 5 | 0 | Normal |
| 5 | 2450 | 1 | 3 | 250 | Normal |

Supplementary Table 2 Chemical composition and pH of the in fluids used in artificial digestion procedure.

| **Composition [mg/mL]** | | | | |
| --- | --- | --- | --- | --- |
| **Saliva, pH 6.4** | | **Gastric Juice, pH 2.0** | **Intestinal Juice, pH 7.5** | |
| 1.667 NaCl | 0.5 CaCl_2_·H_2_O | 4.143 NaCl | 0.3 NaCl | 0.5 CaCl_2_·H_2_O |
| 0.5 NaSCN | 0.33 Ureate | 1 KCl | 0.5 NaSCN | 0.2 MgCl_2_·6 H_2_O |
| 1.833 Na_2_SO_4_ | 0.033 Urea | 0.386 KH_2_PO_4_ | 0.5 NaHCO_3_ | 9 Pancreatin |
| 0.5 NaHCO_3_ | 0.833 α-Amylase | 4.286 Mucin | 0.3 Trypsin | 9 Bile extract |
| 1.5 KCl | 2.5 Mucin | 1.429 Pepsin | 0.3 Ureate |  |
| 2.0 KH_2_PO_4_ |  | HCl for titration | NaHCO_3_ (s) for titration | |


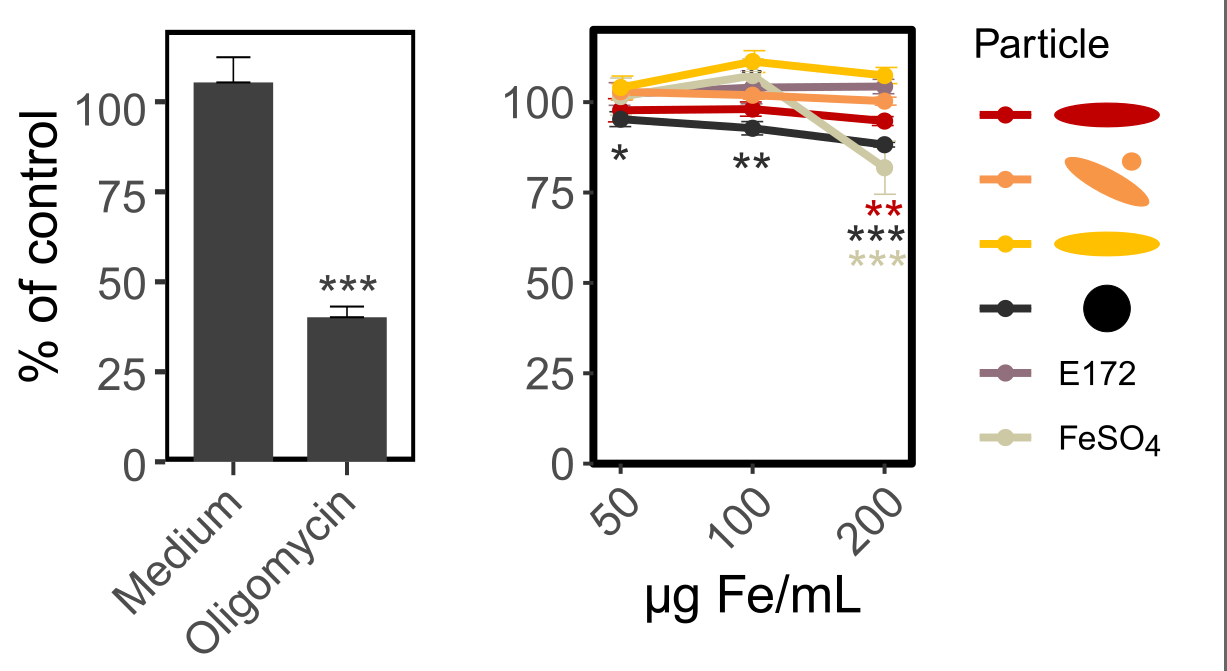


Supplementary Figure 1 Effect of iron oxide nanoparticles on the ATP content of HepaRG cells. Since Caco-2 cells interfered with the assay, only HepaRG cells were tested. The assay was conducted after 24 h of incubation with the respective test substances. Mean ± sd, n=3, statistical analysis was done with one-way ANOVA followed by Dunnett’s test (* p<0.05, ** p<0.01, *** p<0.001).


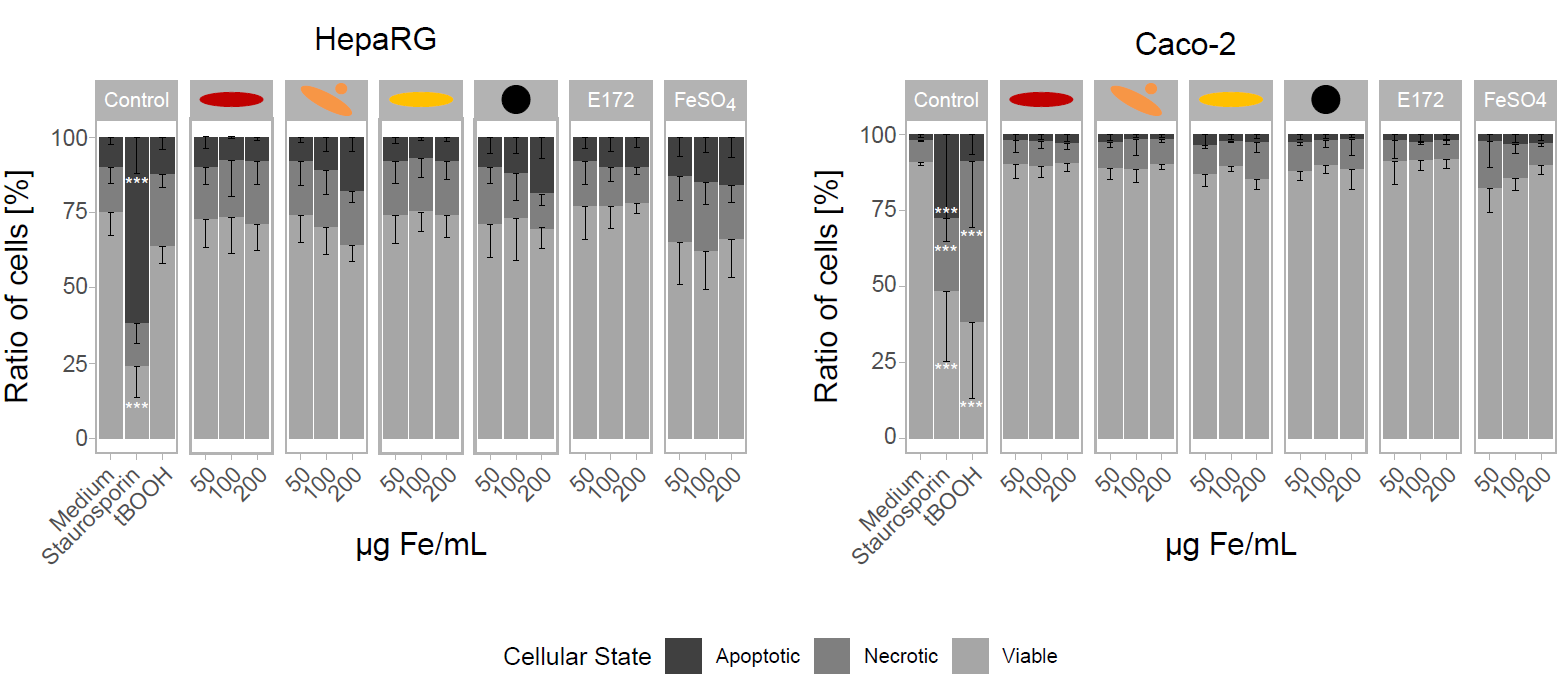


Supplementary Figure 2 Proportion of apoptotic, necrotic and viable cells after treatment with iron oxide nanoparticles. Differentiated HepaRG and Caco-2 cells were incubated with 50 µg Fe/mL for 24 h. 2 μM Staurosporin and 100 µM tert-butyl-hydroxy peroxid (tBOOH) served as positive controls for apoptosis and necrosis/late apoptosis, respectively. After that, they were trypsinated at stained with AnnexinV/7-AAD and analyzed using the FACS BD Accuri C6 (BD, Heidelberg, Germany). Mean ± sd, n=3, statistical analysis was done with one-way ANOVA followed by Dunnett’s test (* p<0.05, ** p<0.01, *** p<0.001).


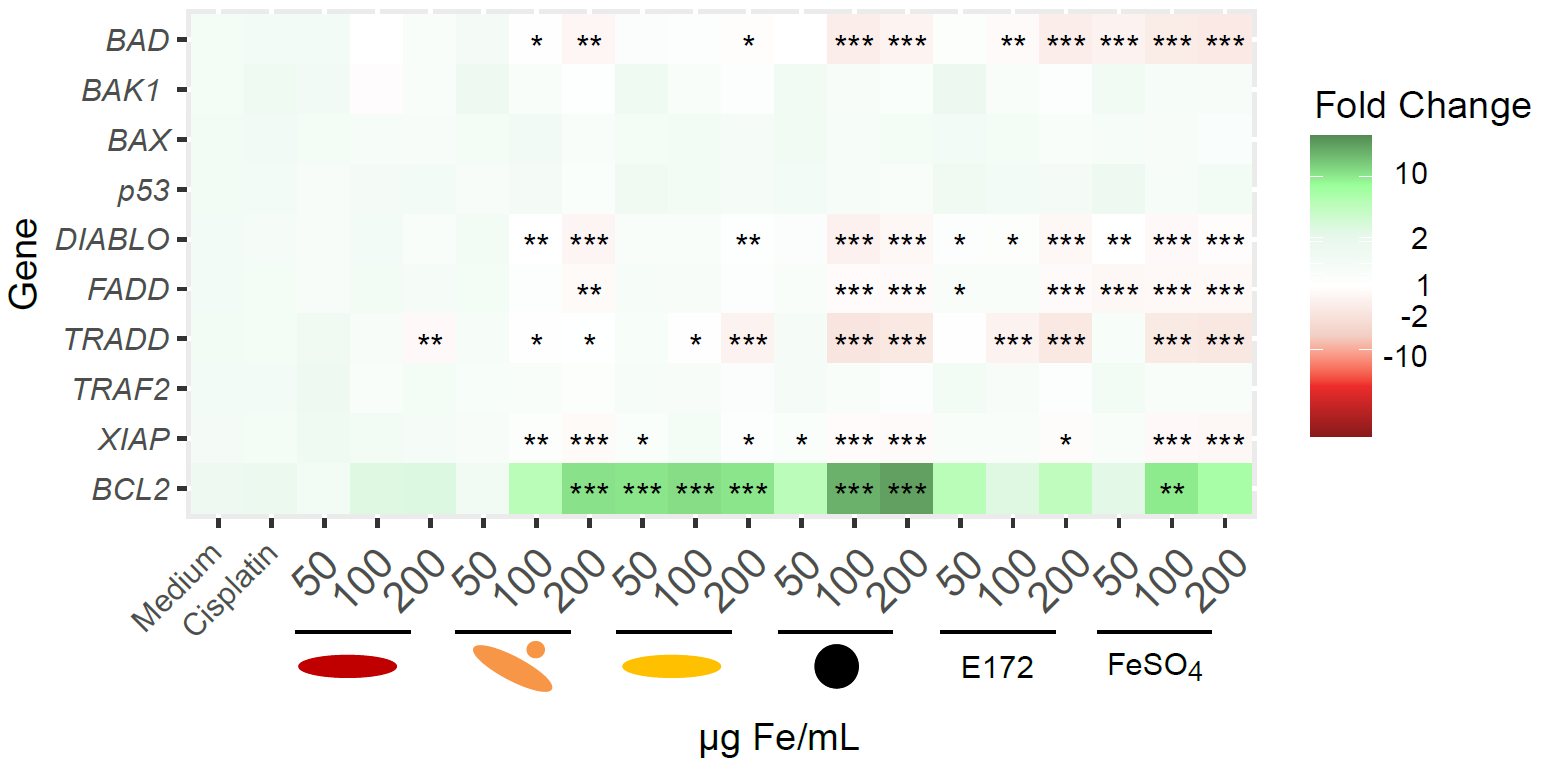


Supplementary Figure 3 Gene expression analysis of genes related to apoptosis. Differentiated HepaRG cells were treated with 50 µg Fe/mL for 24 h. After cell lysis, RNA extraction and cDNA reverse transcription, qRT-PCR was used to quantify the fold change of gene expression with the delta delta-ct method. Red indicates a decrease in transcripts, while green shows transcript upregulation. Mean ± sd, n=3, statistical analysis was done with one-way ANOVA followed by Dunnett’s test (* p<0.05, ** p<0.01, *** p<0.001).
